# What is the impact of social support on self-rated health and depressive symptoms in university students: a survey study in two German universities

**DOI:** 10.1186/s12889-025-23362-3

**Published:** 2025-07-03

**Authors:** Mira Tschorn, Kristin Koller-Schlaud, Darlene Heinen, Sarah Jane Böttger, Julia Seiffert, Veronika Esipova, Timm Seegert, Amanda Sophie Voss, Bernd Förstner, Michael Rapp, Jan C. Zoellick

**Affiliations:** 1 Social and Preventive Medicine, Department of Sports and Health Sciences, University of Potsdam, Am Neuen Palais 10, 14469 Potsdam, Germany; 2 German Center for Mental Health (DZPG), partner site Berlin/Potsdam, Potsdam, Germany; 3 Department of Psychiatry, Psychotherapy and Psychosomatics, University Hospital Ruppin-Brandenburg, Brandenburg Medical School, Fehrbelliner Straße 38, 16816 Neuruppin, Germany; 4 Academic Sports Center, Student Health Management, University of Potsdam, Am Neuen Palais 10, 14469 Potsdam, Germany; 5 Institut und Poliklinik für Arbeits-, Sozial- und Umweltmedizin, Friedrich-Alexander-Universität Erlangen-Nürnberg, Henkestraße 9-11, 91054 Erlangen, Germany; 6 Insitute of Medical Sociology and Rehabilitation Science, Charité – Universitätsmedizin Berlin, corporate member of Freie Universität Berlin and Humboldt-Universität zu Berlin, Charitéplatz 1, 10117 Berlin, Germany

**Keywords:** Self-rated health, Mental health, Depressive symptoms, Social support, University students

## Abstract

**Background:**

Mental health problems have been shown to be highly prevalent among university students with symptoms of stress, depression and anxiety exerting a substantial negative impact. Factors such as gender and social support have been considered important factors for mental and self-rated health. The present study aims to report on the mental and self-rated health of students at two universities within the Berlin-Potsdam metropolitan area (Germany) and to investigate potential associations between social support, self-rated health and depressive symptoms in these two samples.

**Methods:**

Students from two universities and different faculties participated in an online-survey reporting on their self-rated health, depressive symptoms and social support. To analyse differences between the two samples we conducted chi square tests for categorical variables, t-tests for continuous variables and Mann-Whitney U tests for non-normally distributed continuous variables. We employed multivariate linear regression models for the two samples separately to investigate the association of the two health indicators with social support while accounting for relationship status, living situation (living alone yes/no) and gender.

**Results:**

31% of students (27–36% in the two samples) reported depressive symptoms above the clinically relevant threshold. Increased depressive symptoms (β=-0.317, *p* <.001; β=-0.326, *p* <.001) and lower overall health (β = 0.222, *p* =.003; β = 0.176, *p* =.008) were associated with fewer social support in both samples. Female gender was associated with higher depressive symptoms (β = 0.207, *p* <.001) and lower overall health (β=-0.231, *p* <.001) in only one of the samples, while gender did not have a significant effect in the other sample. Relationship status and living situation were not found to have a significant effect.

**Conclusions:**

With roughly a third of students reporting depressive symptoms at both study sites, our data supports the notion that mental health difficulties are a critically relevant topic in the university context. The association of social support with depressive symptoms and overall health in both samples with evidence for gender-specific effects underline the importance of considering social and gender aspects when understanding university student mental health in this particular phase. Our findings contribute to the understanding of relevant factors with the potential to positively impact university students’ mental health.

## Introduction

Students commencing their university education enter a transition phase. During that time, they are facing personal and developmental challenges including self-identity formation, intellectual and social development in the work context, high degrees of self-organization and autonomy as well as settling into the institutional context of academia [1, 2]. For many university students, this transition phase is characterized by separation from home due to a change of residence, relationship stressors, increased demands on time management skills and overall high levels of psychological stress [3, 4]. Moreover, the mean age for students starting their university education is known to be a peak risk period for the onset of mental disorders such as affective disorders [5]. University students can thus be considered a particularly vulnerable population regarding mental illness.

According to the German health insurance company “Techniker Krankenkasse” [6] as well as the Federal Ministry of Education and Research [7], mental health problems are particularly prevalent among university students with stress, depression and anxiety symptoms exerting notably negative effects. Their findings also reveal that in 2023 nearly two-thirds of students in Germany rated their overall health as good or very good, while approximately one-tenth rated it as fair or poor. Moreover, the proportion of students reporting negative health has more than tripled since 2015 (3% in 2015, 10% in 2023). A study conducted by another German health insurance company “BARMER” [8] reported that in 2015 17% of their student clients were affected by a professionally diagnosed mental illness. 5% of all students were diagnosed with an affective disorder, with depressive episodes being the second most common diagnosis affecting 4% of all students. The prevalence of diagnosed depressive disorders in young adults (general population aged 18 to 25) has increased by 66% in recent years according to “BARMER” data [8]. The reported prevalence of self-reported depressive symptoms varies depending on the educational institution and the methodology of data collection. However, the estimates suggest a range of between 20 and 33% of university students being affected by depressive symptoms, a proportion notably higher than that observed in age-matched non-student populations or in the general German population. Some studies suggest that women might be more affected than men [9, 10], however, systematic reviews [11] and meta-analyses remain inconclusive [12].

The rising prevalence of mental health issues among university students underscores the broader consequences of psychological distress. These consequences do not only impact academic performance and retention rates [13, 14], but also extend into long-term challenges in personal well-being, workplace productivity and societal economic costs [15, 16]. University students affected by poor mental health also report more difficulties in coping with study demands and report lower study satisfaction [17].

At the same time, psychosocial and sociodemographic factors also play a significant role in influencing university students’ mental and subjective well-being [11, 18, 19]. Studies have shown that loneliness and social isolation are related to poorer mental health [11]. Regarding social support, feelings of loneliness and social support among peers emerged as the strongest predictor of poor mental health [20]. Students with lower social support, particularly those who are single or non-parents, were reported to exhibit higher levels of depressive symptoms, a trend that was particularly pronounced during the COVID-19 pandemic [19]. Relationship status also plays a key role in mental well-being, with married individuals generally reporting better mental health compared to those who are single, divorced, or widowed [21, 22]. Cohabitation and intimate partnerships offer similar protective benefits, presumably due to enhanced social and financial support [23, 24]. Further, studies have shown that having a partner is not associated with overall health, whereas living with parents during academic terms was linked to better health outcomes [25]. Aside from psychosocial and sociodemographic factors, perceived psychological distress in university students has been linked to study conditions and contextual factors such as study demands or qualification potential [20]. The field of study also seems to play a role; Students of linguistics and cultural studies reported high levels of depressive and anxiety symptoms [16], students of veterinary medicine, law and physics reported being particularly affected by stress [26] and students of medicine report lower levels of depressive symptoms compared to students of other fields of study [27]. Similarly, a meta-analysis found a higher weighted mean for elevated depressive symptoms in heterogenous student samples (36%), compared to medical student studies (26%) [28]. However, a meta-analysis comparing depression prevalence in medical versus non-medical students found slightly but not significantly higher prevalence rates in non-medical students [12].

Lastly, the COVID-19 pandemic and the governmental responses could have contributed to a deterioration of mental health among university students. University closures and the shift to online learning led to increased isolation and lifestyle changes, while lockdowns and other restrictions negatively affected university students’ health [29, 30]. This period of prolonged stress and reduced social interactions likely contributed to a decline in students’ overall health perceptions, as they struggled with disrupted routines, financial uncertainties and fewer support networks [29, 30]. Gewalt et al. [31] surveyed 917 students at eight universities in the southwest of Germany in May and June 2020 and found that 53% of students reported a decline in mental health, with 9% indicating a significant worsening compared to pre-pandemic levels. Using a longitudinal design, Werner et al. [32] reported increases in depression, but not anxiety or somatic complaints in 442 students at the University Mainz, Germany, in June 2020 compared to pre-pandemic baseline levels from June/August 2019. Holm-Hadulla et al. [27] surveyed 2,135 students from Heidelberg University, Germany, from May to June 2021 and found that 72% of students reported having experienced significant impairments in well-being and 76% exhibited symptoms of at least one mental illness, with 42% showing depressive symptoms.

In summary, the transition to university life poses significant challenges for university students, including heightened risk for their mental health. The increasing prevalence of stress, anxiety and depression in this group underscores the need for targeted interventions. The present study aims to report on the mental health of students at two universities and to investigate potential associations between social support, self-rated health and depressive symptoms in the two samples. The study seeks to address the following research questions:

What is the prevalence and level of depressive symptoms, social support and self-rated health among university students at University of Potsdam and Charité – Universitätsmedizin Berlin, two institutions with markedly different academic profiles?

Is there an association between perceived social support and depressive symptoms or subjective health in the two samples?

Are there differences in levels of depressive symptoms, self-rated health and perceived social support between students from the two universities?

## Methods

We combined the data of two separate data collections at two universities in the Berlin-Potsdam metropolitan area (Germany) to investigate similarities and differences in the two samples and gather information about potential influencing factors on these associations such as gender and relationship status.

Data for the present analyses were collected as part of two separate studies at the University of Potsdam (UP) and at Charité – Universitätsmedizin Berlin, respectively. While Charité primarily offers programs in medicine, dentistry, midwifery, and nursing – with a strong focus on medical education – the University of Potsdam provides a broad range of study programs across six faculties (Faculty of Law, Faculty of Humanities, Faculty of Human Sciences, Faculty of Business, Economics and Social Sciences, Faculty of Mathematics and Natural Sciences, and Faculty of Digital Engineering). Accordingly, the academic profiles of the two universities differ substantially.

For the UP sample, the study was approved by the local ethics committees of the University of Potsdam in accordance with the Declaration of Helsinki (No. 06/2016 with the amendments 62/2019, 59/2023 and 100/2023). For the Charité sample, data collection was part of a multicentre project approved by the local ethics committee of the Friedrich-Alexander University of Erlangen-Nuremberg in accordance with the Declaration of Helsinki, which rated that no detailed examination would be necessary for the study (vote 21-393-ANF from 21 November 2021). All participants provided their written informed consent prior to participation in the study.

### Participants and data collection

Data collection for the UP sample took place over a two-month period during the winter term 2023/2024, from 11 December 2023 to 5 February 2024, and was advertised by the local Student Health Management via internal mailing lists of the Zentrum für Hochschulsport (Centre for University Sports, *N* = 15,000), e-mail, flyers, posters and personal contacts within the university. 185 participants from 6 faculties completed the online survey within the university survey system Umfragen.UP (based on SoSci Survey), which was accessed through a link or QR code on the study invitation (1.23% response rate with respect to the mailing list). The six faculties at the UP were: Faculty of Law, Faculty of Humanities, Faculty of Human Sciences, Faculty of Business, Economics and Social Sciences, Faculty of Mathematics and Natural Sciences and Faculty of Digital Engineering.

Data collection for the Charité – Universitätsmedizin Berlin sample took place over a three-month period during the winter term 2023/24 from 29 November 2023 to 1 March 2024. The online study invitation was send out via mailing lists from the dean of study and further advertised by the local student union. Approximately 5,500 students were enrolled in the programs medicine, dentistry, midwifery and nursing at the Medical Faculty in 2023, of which 296 participants completed the online survey (5% response rate). A-priori power analyses or sample size calculations were not carried out as the analyses presented in this article were based on data sets of two independent exploratory cross-sectional survey studies.

### Measures

This section describes the assessments used in the two samples to collect the data analysed for the present study. The data analysed for the present article were selected based on the overlap of assessments at Charité- Universitätsmedizin Berlin and University of Potsdam.

### Sociodemographic variables

Sociodemographic information including gender (female, male, diverse, not specified), age, partnership status, family and living situation were assessed in both samples. To allow for comparison of the two samples, socio-demographic information on family status and living situation were combined into the following modified dichotomous variables: partnership status (yes/no), having children (yes/no) and living alone (yes/no).

### Social support

Social support is a psychosocial resource that is provided through interpersonal relationships and the networks we cultivate [33]. There is significant variation in how social support is defined and measured [34–36]. Moreover, the conceptualization and measurement of social support also varies according to disciplinary perspectives [37]. In the following section, we examine various facets of social support, each of which is emphasized differently across disciplines, conceptual frameworks, and measurement tools.

The structural aspect of social connections provides a descriptive overview of an individual’s social network and is often regarded as social integration [38, 39]. From a psychological perspective, social support most commonly refers to functional and qualitative aspects of social relationships [39]. The functional aspect of social support is typically assessed through instrumental, emotional or financial dimensions. Two key aspects of functional support—instrumental and emotional—are frequently linked to overall mental health and are particularly associated with depression and depressive symptoms [35, 40, 41]. The subjective evaluation by the individual of whether such support is available and perceived as sufficient plays a crucial role in influencing health outcomes. In the present study social support was assessed differently in the two analysed samples. For assessing social support in the UP sample, item 2 of the Oslo-3 [42, 43] scale was used: ‘How much interest and concern do people show in what you are doing?’ (possible answers were: ‘a lot of concern and interest’, ‘some concern and interest’, ‘uncertain’, ‘little concern and interest’ and ‘no concern and interest’). The Oslo-3 scale has been validated in a German population sample (*N* = 2,524), showing a one-factor structure and an internal consistency of Cronbach’s α = 0.64, which is acceptable for ultra-brief instruments. Item 2 is an integral part of this validated scale and contributes to its overall construct validity [35]. In the Charité sample, the 15-item Personal Resource Questionnaire (PRQ 2000) was used to assess the perceived level of social support [44]. The 15 items, e.g., “I know that others appreciate me as a person” are answered on a 7-point Likert scale ranging from strongly disagree [1] to strongly agree [7]. A common mean for the entire scale then was calculated with higher scores indicating higher levels of perceived social support. The PRQ-2000 has shown high internal consistency, with Cronbach’s alpha ranging from α = 0.91–0.93 across different populations. The construct validity has been supported by significant positive correlations with psychological well-being and health-promoting behaviors [44]. We recognize that these instruments do not include a specific assessment of social support and lack an explicit subjective evaluation of social support, particularly in the Oslo-3 single item. However, as these instruments are self-assessments, it is likely that they contain a subjective component in which participants’ personal assessment of their social integration influences their response. The Oslo-3 single item assesses structural aspects of an individual’s social networks that can be understood as social integration as well as facets of emotional support, as it reflects the feeling of being cared for and valued by others. Nevertheless, since our measures do not differentiate specific aspects of social support and since the PRQ 2000 is a broader assessment than the Oslo-3 single item measure, we discuss the limitations resulting from the use of a comparison of two different instruments in the limitations section. Despite the remaining imprecision of these instruments with regard to the operationalization of social support, we refer to the constructs measured here as social support in line with the terminology used by the instrument developers.

### Health outcomes

**Depressive symptoms** were assessed using the two-item version of the Patient Health Questionnaire (PHQ-2; [45]), a brief self-report screening instrument for depression that is comprised of the first two items of the longer version PHQ-9. The two items assess depressed mood and anhedonia over the last two weeks on a 4-point scale. A validation study of the PHQ-2 reported robust screening accuracy for major depressive disorder with a sensitivity of 83% and specificity of 92% at a threshold score of ≥ 3 [46]. The scale also demonstrated strong concurrent validity, as indicated by a high correlation with the PHQ-9 (*r* =.83) and an overall diagnostic accuracy (AUC = 0.90).

**Self-rated health** (SRH) was assessed using two slightly different versions of the internationally established indicator of general health. In accordance with the wording in the socio-economic panel [47], participants in the UP sample were asked “How would you describe your current health?”. The response options were ‘very good’ (= 1), ‘good’, ‘satisfactory’, ‘poor’ and ‘bad’ (= 5) which were then dichotomized into the categories ‘rather good’ (very good and good) and ‘rather poor’ (other three categories).

In accordance with a recommendation of the World Health Organization (WHO) [48], participants in the Charité sample were asked: ‘How is your health in general?’. The participants responded using a five-point scale (very good = 1, good, average, poor, very poor = 5), which was combined into the categories of ‘very good/good’ and ‘average/bad/very bad’ for the present analyses.

Since the wording in the two versions only differed slightly, we were able to analyse ‘positively perceived subjective health’ as defined by the nationwide health monitoring (‘very good’ or ‘good’ SRH) for both versions of SRH assessments.

Despite the slight difference in wording, both SRH items assess the same underlying construct. Schnittker and Bacak [49] showed that SRH strongly predicted mortality, with odds ratios up to 3.13 for poor versus very good self-rated health and that this predictive power had increased over time. The two SRH items in the Charité sample [50] were therefore recoded and harmonized with the UP sample to ensure comparability between samples.

### Data analysis

All statistical analyses were performed using IBM SPSS Statistics version 29 [51].

To analyse differences between the two samples, we conducted chi square tests for categorical variables, t-tests for continuous variables (including Cohen’s d and Cramer’s V as effect size for t-tests and chi square tests) and Mann-Whitney U tests for non-normally distributed continuous variables. Results are reported as statistically significant at a level of p⋜ 0.05. Confidence intervals (CIs) are reported at the 95% confidence level. Cohen’s d values of 0.2, 0.5, and 0.8 were interpreted as small, medium, and large effect sizes, respectively. Cramer’s V values of 0.1, 0.3, and 0.5 were interpreted as small, moderate, strong associations. Analyses were conducted separately for each university, as the academic profiles of the institutions differ markedly, with Charité focusing on health-related disciplines (primarily medicine), and the University of Potsdam offering a broad spectrum of study programs across six faculties. These structural differences may influence both the health outcomes of students and the relevance of the psychosocial predictors under study. Additionally, the assessment instruments for some variables were not fully identical across the two samples, which further supported the decision to analyze the data separately.

### Multivariate analyses

Since different measures were used to assess social support in the two samples, multivariate linear regression models were analysed separately for the two samples to investigate the association of the two health indicators (SRH and depressive symptoms) with social support while also accounting for relationship status (yes/no), living situation (living alone yes/no) and gender (f, m). Due to the small cell sizes of the participants who indicated ‘diverse’ or ‘no response’ for the gender variable, the regression analyses had to be carried out excluding these cases. However, all regression analyses were repeated without the gender predictor and with the full sample to analyse potential effects of the exclusion of these subjects, which did not change the results.

### SRH - Assumptions for the regression models

We found no evidence of significant multicollinearity with variance inflation factors (VIF) below 1.066 (UP) or 1.132 (Charité) for all predictors (gender, social support, relationship status, living alone). In addition, the average VIF for all predictors was 1.040 (UP) and 1.078 (Charité) and the tolerance statistics were above 0.938 (UP) and above 0.883 (Charité) [52–54].

### Depressive symptoms- assumptions for the regression models

We found no evidence of significant multicollinearity with variance inflation factors (VIF) below 1.066 (UP) or below 1.135 (Charité) for all predictors (gender, social support, relationship status, living alone). In addition, the average VIF for all predictors was 1.040 (UP) and 1.080 (Charité) and the tolerance statistics were above 0.938 (UP) and above 0.881 (Charité) [52–54].

Since there was evidence for heteroskedasticity (Breusch-Pegan-Test *p* <.05) within the model analysing PHQ-2 with the predictors gender, social support, relationship status and living situation for the Charité sample, we report p-values for heteroscedasticity-consistent parameters for this specific model [55].

## Results

Sociodemographic and clinical characteristics of the study sample are depicted in Table 1 both for the entire sample of 428 participants and for the two samples (UP and Charité) separately. The mean age of the participants was 24 years (range 17.4–57 years) and 80% were female. The two university samples differed regarding their relationship status with a higher rate (52% vs. 41%) of participants in a relationship in the Charité sample (χ^2^ [1] = 6.416, *p* =.011).

**Table 1:** Sample characteristics

Variables	Overall Sample	University of Potsdam	Charité - Universitätsmedizin Berlin	*p*
*N*	428	185	243	
Gender Female (n, %) Male (n, %) Diverse (n, %) Missing (n, %)	312, 72.9% 106, 24.8% 5, 1.2% 5, 1.2%	136, 73.5% 45, 24.3% 1, 0.5% 3, 1.6%	176, 72.4% 61, 25.1% 4, 1.6% 2, 0.8%	0.167
Age (mean in years, ±SD)	24.17, ± 1.64^1^	24.56, ± 5.98	23.86, ± 4.63^1^	0.180
Children (yes, %)	28, 6.5%	11, 5.9%	17, 7.0%	0.663
In a relationship (yes, %)	185, 47.2%^2^	75, 40.5%	127, 52.3%^2^	**0.011**
Living alone (yes, %)	126, 29.4%	62, 33.5%	64, 26.3%	0.107
Social support, PRQ (mean, ±SD)	-	-	5.83, ± 1.01^2^	-
Social support, Oslo (item 2) (mean, ±SD)	-	3.37, ± 0.81	-	-
PHQ2 (mean, ±SD)	2.10, ± 1.64^3^	2.26, ± 1.76	1.98, ± 1.53^3^	0.082
PHQ2 above cut-point (n, %)	131, 31.0%^3^	66, 35.7%	65, 26.7%^3^	0.065
SRH: harmonized scales) (mean, ±SD)^5^	2.47, ± 0.86^4^	2.37, ± 0.81	2.54, ± 0.88.3^4^	**0.037**
SRH: positively perceived subjective health (n, %)	293, 68.5%^4^	115, 62.2%	178, 78.3%^4^	**0.010**
Depression or SRH above cut-point	194, 45.3%^4^	99, 53.5%	95, 39.1%^4^	**0.003**

Note. SD = standard deviation, PRQ = Personal Resource Questionnaire, PHQ = patient health questionnaire, SRH = self-rated health, ^1^ missing *n* = 11, ^2^ missing *n* = 3, ^3^ missing *n* = 5, ^4^ missing *n* = 2, ^5^ higher scores indicate worse SRH

### Bivariate analyses

Students in the Charité sample reported better subjective health: On average, students at Charité reported better subjective health (M = 2.54, ± 0.88.3) compared to students at UP (2.37, ± 0.81; t(424) = 2.096, *p* =.037, d = 0.205) and students at Charité more often reported positively perceived subjective health (78%) compared to students at UP (62%; χ^2^ [1] = 6.668, *p* =.010, V = 0.125). Also, the rate of students who either reported elevated depression scores or who did not report positively perceived subjective health was lower in the Charité sample (39%) compared to the students at UP (54%; χ^2^ [1] = 8.812, *p* =.003, V = 0.143). All reported differences were small in regards to their effect sizes.

### Depressive symptoms: multivariate regression analyses

In the UP sample, higher depressive symptom scores were associated with lower social support (β=-0.317, CI=-0.459 – − 0.175, *p* <.001, see Table 2) and were not associated with gender, relationship status or living alone (all *p* >.12). Similarly, in the Charité sample, higher depression scores were linked to lower social support (β=-0.326, CI=-0.451 – − 0.201, *p* <.001) and no association was found with relationship status or living alone (all *p* >.12). In this sample however, gender was linked to depressive symptoms, with higher depression scores in female students (β = 0.207, CI = 0.085 – 0.239, *p* <.001).

**Table 2:** Prediction of depressive symptoms (PHQ-2) and self-rated health in two university samples

	University of Potsdam *N* = 181^1^			Charité – Universitätsmedizin Berlin *N* = 229^1^		
Variables	Standardized beta (95% CI)		*p*	Standardized beta (95% CI)		*p*
**Depressive symptoms**						
Gender (male/female)	− 0.043	(-0.183 – 0.097)	0.545	0.207	(0.085 – 0.329)	**< 0.001**
Social support^1^	− 0.317	(-0.459 – − 0.175)	**< 0.001**	− 0.326	(-0.451 – − 0.201)	**< 0.001**
Relationship (yes/no)	− 0.060	(-0.203 – 0.083)	0.409	0.108	(-0.020 – 0.236)	.121^2^
Living alone (yes/no)	− 0.111	(-0.253 – 0.031)	0.124	0.021	(-0.105 – 0.147)	.762^2^
**Self-rated health**						
Gender (male/female)	0.005	(-0.133 – 0.143)	0.941	− 0.231	(-0.357 – − 0.105)	**< 0.001**
Social support^1^	0.222	(0.077 – 0.367)	**0.003**	0.176	(0.045 – 0.306)	**0.008**
Relationship (yes/no)	0.126	(-0.022 – 0.275)	0.095	− 0.045	(-0.174 – 0.090)	0.532
Living alone (yes/no)	− 0.012	(-0.155 – 0.132)	0.871	− 0.044	(-0.175 – 0.087)	0.508

Note. CI = Confidence Interval, University of Potsdam: depressive symptoms *R*^*2*^ = 0.118, self-rated health *R*^*2*^ = 0.074; Charité - Universitätsmedizin Berlin: depressive symptoms *R*^*2*^ = 0.157, self-rated health *R*^*2*^ = 0.091, ^1^ Reduced *N* due to exclusion of “diverse” and “missing” cases regarding gender for the multivariate models, ^2^ p values corresponding to heteroscedasticity-consistent parameters

### Subjective health status: multivariate regression analyses

In the UP sample, higher (i.e. better) SRH was associated with higher social support (β = 0.222, CI = 0.077 – 0.367, *p* =.003, see Table 2) and was not associated with gender, relationship status or living situation (all *p* >.09). Similarly, in the Charité sample, higher SRH scores were linked to higher social support (β = 0.176, CI = 0.045 – 0.306, p = < 0.008) and no association was found with relationship status or living situation (all *p* >.50). In this sample, gender was linked to SRH with higher (i.e. better) SRH in male students (β=-0.231, CI=-0.357 – − 0.105, p = < 0.001).

Excluding gender as a predictor and analysing the full sample including students who indicated ‘diverse’ or ‘no response’ for the gender variable did not alter the present results.

## Discussion

The present study aimed to report on the mental health of university students at two German universities and to investigate potential associations between social support, self-rated health, and depressive symptoms in these two samples. Our study found that 31% of all surveyed students (36% at UP and 27% at Charité) reported depressive symptoms above the clinically relevant threshold. When exploring factors contributing to depressive symptoms, our results revealed significant associations with social support and gender. Specifically, increased depressive symptoms and lower overall health were significantly associated with reduced social support in both samples and with female gender in the Charité sample. Neither romantic relationships nor living alone were predictive of the outcomes in our sample. Comparing the two university samples, our data showed differences in relationship status, reported subjective health, depression scores and positively perceived subjective health. These differences may, at least in part, be attributed to structural and contextual distinctions between the two universities. Charité – Universitätsmedizin Berlin primarily offers medical and health-related programs and is located in Germany’s largest urban center, while the University of Potsdam comprises a broad spectrum of disciplines and is situated in a smaller, less urban environment. Prior research suggests that such differences in academic profiles and regional contexts are associated with distinct student characteristics and experiences, including personality traits and educational engagement [56]. These institutional and contextual factors may influence students’ mental health and perceived social support, and thus offer an explanation for the variation observed between the two samples.

The prevalence of depressive symptoms in university student samples has been quantified at 31% (95%-CI: 30-31%) across 24 studies with 48,650 participants [28], and at 28% (95%-CI: 24-32%) for medical students in particular across 72 studies with 62,728 participants [12] which corresponds with our findings. We used the PHQ-2 as a two-item screener for depression and found a similar prevalence as in other studies, e.g. from France reporting a prevalence of 23% of students with elevated depression scores [57]. In contrast, studies from health insurance companies regularly report a depression prevalence of 4–7% using claims data rather than symptom screeners [8]. This methodological difference demonstrates how prevalence rates can vary depending on the type of data collection used, such as self-reports versus clinical assessments.

We observed gender differences only in the Charité sample, but not in the UP sample which aligns with the inconsistency in results reported in meta-analyses. In the meta-analysis by Ibrahim et al.’ [28] studies reported a higher prevalence in women (30%) compared to men (25%). In contrast, Puthran et al. [12] found in their meta-analysis that the difference between women (32%) and men (24%) was not statistically significant. These inconsistencies in university student samples are surprising, as gender differences with women reporting worse mental health than men are well-documented in population-representative adult samples [58] and among adolescents [59]. Qualitative research might be needed to understand the peculiarities in student samples in contrast to other cohorts.

In our study, we found associations between lower social support, increased depressive symptoms and lower self-rated health in both samples consistent with previous studies showing the importance of social support on university student health [60, 61] even though cohort studies did not find perceived social support to impact depression [62]. Although perceived social support is often viewed as a protective factor, a significant direct impact on depression risk may not always be observed. Nevertheless, there could be a potential for universities to positively impact student health by strengthening peer networks and institutional support systems. These associations may be understood in the context of the stress-buffering hypothesis, which suggests that social support mitigates the negative effects of stress on mental and physical health by shaping how individuals perceive and cope with stressors [34]. Recent research highlights that **perceived social support** reduces psychological distress by lowering perceived stress levels [63]. In addition, biological pathways—such as the regulation of the HPA axis—may explain how social support influences general health [64]. The associations found in our study may thus reflect both psychological and physiological mechanisms of stress regulation, which are particularly relevant in the student context.

Despite the positive effect of social support in our study, neither partnerships nor living alone or together with others as two direct forms of social support influenced SRH in our samples. These findings stand in contrast to the body of literature that identified partnerships as a protective factor against depression [18, 65]. Many studies address partnerships in later life [21, 22] when stable romantic partnerships might gain importance compared to the university study phase with its transitional nature. Corresponding to our findings, Farrer et al. [61] also found that romantic relationships did not significantly influence depression risk. Instead of partnerships, peer-/university groups might provide the social support that protects students against poor SRH [60]. We assume that it is the quality of romantic relationships, rather than their mere existence, that matters in determining whether they further enhance feelings of social support. Empirical evidence also suggests that it is not relationship status per se, but rather the perceived quality and emotional support within the relationship that is influencing mental health [66, 67]. Regarding living circumstances, Mikolajczyk et al. [25] found that living with parents during the term was associated with better mental health, but relationship status did not have an effect on mental health outcomes. In contrast, Freire et al. [68] observed no significant association between living situation and SRH. Farrer et al. [61] reported that relocating to university increased the risk of depression, highlighting the importance of stability during transitions. Further research is necessary to better understand the mechanisms through which social support can positively impact university student mental health. Further understanding could assist in designing and implementing effective interventions to support university students not only academically but also emotionally and socially.

As for differences between the samples of the two institutions in our study, we can only speculate about the reasons for the finding that UP students form fewer romantic relationships and report lower self-reported health. The UP sample in the present study reported depressive symptoms more frequently. These differences could point to the possibility that variations in cultural contexts and mental health support systems between regions or universities may influence the prevalence of depressive symptoms reported by students. In this context, the socioeconomic status (SES) of students could also play a role. Although university students, on average, come from more privileged socioeconomic backgrounds compared to the general population, it is important to recognize that significant socioeconomic inequalities exist within the student body itself. Previous research has shown that university students from socially disadvantaged backgrounds face greater financial burdens, have poorer health indicators and have limited access to academic and social resources [69] and that social inequalities among university students have an impact on their mental health [70]. Further research is needed to understand regional differences and peculiarities.

### Limitations

Our study is limited by the sole use of self-report assessments and convenience sampling as design choices. Recruiting for a survey on study demands and mental health might have appealed more to those burdened by study demands and mental health difficulties. Such a selection bias in our convenience samples might explain the relatively high number of students reporting depressive symptoms. However, it does not explain the strong gender effect at Charité. Self-reports are naturally limited by selection bias and subjectivity.

The response rate in both study samples was low (Charité: 5%, UP: 1.23%), which limits the representativeness of the data and increases the likelihood of selection bias. It is possible that students experiencing greater psychological distress or stronger health-related concerns were more likely to participate in the survey. This could partially explain the higher proportion of depressive symptoms observed in the UP sample. However, low response rates are a known challenge in student surveys, particularly when dealing with sensitive topics like mental health and when no direct incentives are provided [71, 72]. While such limitations constrain the generalizability of prevalence estimates, they do not necessarily invalidate the observed associations within the sample. Future research should aim to increase response rates through targeted recruitment strategies or incentives and consider longitudinal or mixed-methods designs to better account for selection effects and their impact on mental health outcomes.

The study uses a cross-sectional design that cannot determine causal relationships between the variables studied. In addition, differences in data collection periods and measures between the two universities may have led to systematic differences in reported symptoms and perceived social support. These differences may affect the comparability of the results.

Furthermore, the operationalization of social support in our study presents certain limitations. While we refer to both instruments used in our study as measures of social support in line with the terminology of the instrument developers, they capture different facets of the construct. The Oslo-3 single item used in the UP sample primarily reflects structural and emotional aspects of social relationships, while the PRQ 2000 used in the Charité sample captures broader, perceived social support. Neither instrument provides a detailed assessment of functional support domains such as instrumental or financial support, and only partially captures subjective appraisal. These differences may limit the depth and comparability of the social support data across samples. Future research should aim to integrate more comprehensive and multidimensional assessments of social support that align more closely with current theoretical frameworks and allow for a more differentiated analysis.

Additionally, a potential overlap between the constructs measured must be acknowledged. For example, the perception of low social support or social integration may reflect underlying feelings of loneliness, which could also influence responses on the PHQ-9, particularly in items assessing mood, energy or interest. This conceptual proximity may partly explain the strength of the observed association between social support and depressive symptoms. Similarly, self-rated health is a subjective measure and may also be affected by psychological states such as loneliness, potentially leading to inflated associations. Future studies should consider using distinct and multi-modal assessments to better disentangle these closely related constructs. In addition, social support in the UP sample was assessed using only a single item from the Oslo-3 scale, which limits the depth and reliability of the construct’s measurement. Single-item measures are more susceptible to measurement error and may not fully capture the multidimensional nature of social support. This further constrains the comparability with the more comprehensive PRQ 2000 measure used in the Charité sample.

The study sample includes students from only two universities (UP and Charité - Universitätsmedizin Berlin). This limits the generalizability of the results to other universities and regions.

Further, the study may not have taken into account all relevant factors that could influence depressive symptoms and perceived social support, such as personal circumstances, financial burdens, cultural differences, migration background or field of study.

This study aimed to capture social support on a general level. Social support should be captured and analysed in more detail in future studies, e.g., whether it is provided by family, friends, peers, or in the form of institutionalized social support at the university. A more nuanced measure of social support would allow for a better understanding of the role and effectiveness of social support and recommendations for how universities can improve student health.

Further research should also include additional protective factors such as resilience, physical activity and the availability of health promotion services.

### Conclusion

Our study assessed health outcomes in students of two German universities with particular focus on depressive symptoms, social support and self-reported health. With roughly a third of university students in both sites reporting depressive symptoms, our results provide further evidence that mental health difficulties are a critically important issue in the university context and underline the need for targeted interventions to promote health in this particular group.

Our findings regarding the association of social support with depressive symptoms and lower overall health in both student samples contribute to the growing body of research pointing to the importance of taking social aspects with their different facets and manifestations into consideration when understanding university student mental health in this particular phase. Further understanding of these factors and their interplay can assist in designing and implementing effective interventions to support university students mental health by assessing and including tailored prevention programs focussing on building social support aspects in the student body. Such programs would address the goal of the German federal government which has obligated universities, as “living environments”, to develop and implement health promotion and prevention services [73, 74]. Considering that our results provided additional evidence for effects of gender, we suggest further research to adequately assess gender-specific aspects to provide further knowledge on the way these aspects should be acknowledged in terms of gender sensitive mental health promotion. Future research should focus on a more nuanced assessment of social support employing longitudinal designs while accounting for gender-specific differences to better understand the mechanisms through which social support can positively impact university student mental health.

## Data Availability

No datasets were generated or analysed during the current study.

## Declarations

**Abbreviations**.

## Abbreviations

BMBF: Federal Ministry of Education and Research
CI: Confidence Interval
COVID-19: Coronavirus Disease 2019
DZPG: German Center for Mental Health (Deutsches Zentrum für Psychische Gesundheit)
ERI: Effort-Reward Imbalance Questionnaire
PHQ: Patient Health Questionnaire
PRQ: Personal Resource Questionnaire
SD: Standard Deviation
SES: Socioeconomic Status
SRH: Self-Rated Health
UP: University of Potsdam
VIF: Variance Inflation Factor
WHO: World Health Organization
